# The basic leucine zipper transcription factor MeaB is critical for biofilm formation, cell wall integrity, and virulence in *Aspergillus fumigatus*

**DOI:** 10.1128/msphere.00619-23

**Published:** 2024-01-29

**Authors:** Yuan Chen, Fei Gao, Xiaojin Chen, Siyuan Tao, Peiying Chen, Wei Lin

**Affiliations:** 1 Nanjing University of Chinese Medicine, Nanjing Drum Tower Hospital, Nanjing, China; 2 Jiangsu Collaborative Innovation Center of Chinese Medicinal Resources Industrialization, Nanjing, China; 3 State Key Laboratory of Bioreactor Engineering, East China University of Science and Technology, Shanghai, China; University of Georgia, Athens, Georgia, USA

**Keywords:** *Aspergillus fumigatus*, cell wall, polysaccharide biosynthesis, biofilm formation

## Abstract

The regulation of fungal cell wall biosynthesis is crucial for cell wall integrity maintenance and directly impacts fungal pathogen virulence. Although numerous genes are involved in fungal cell wall polysaccharide biosynthesis through multiple pathways, the underlying regulatory mechanism is still not fully understood. In this study, we identified and functionally characterized a direct downstream target of SomA, the basic-region leucine zipper transcription factor MeaB, playing a certain role in *Aspergillus fumigatus* cell wall integrity. Loss of *meaB* reduces hyphal growth, causes severe defects in galactosaminogalactan-mediated biofilm formation, and attenuates virulence in a *Galleria mellonella* infection model. Furthermore, the *meaB* null mutant strain exhibited hypersensitivity to cell wall-perturbing agents and significantly alters the cell wall structure. Transcriptional profile analysis revealed that MeaB positively regulates the expression of the galactosaminogalactan biosynthesis and β-1,3-glucanosyltransferase genes *uge3*, *agd3*, and *sph3* and *gel1*, *gel5,* and *gel7*, respectively, as well as genes involved in amino sugar and nucleotide sugar metabolism. Further study demonstrated that MeaB could respond to cell wall stress and contribute to the proper expression of mitogen-activated protein kinase genes *mpkA* and *mpkC* in the presence of different concentrations of congo red. In conclusion, *A. fumigatus* MeaB plays a critical role in cell wall integrity by governing the expression of genes encoding cell wall-related proteins, thus impacting the virulence of this fungus.

**IMPORTANCE:**

*Aspergillus fumigatus* is a common opportunistic mold that causes life-threatening infections in immunosuppressed patients. The fungal cell wall is a complex and dynamic organelle essential for the development of pathogenic fungi. Genes involved in cell wall polysaccharide biosynthesis and remodeling are crucial for fungal pathogen virulence. However, the potential regulatory mechanism for cell wall integrity remains to be fully defined in *A. fumigatus*. In the present study, we identify basic-region leucine zipper transcription factor MeaB as an important regulator of cell wall galactosaminogalactan biosynthesis and β-1,3-glucan remodeling that consequently impacts stress response and virulence of fungal pathogens. Thus, we illuminate a mechanism of transcriptional control fungal cell wall polysaccharide biosynthesis and stress response. As these cell wall components are promising therapeutic targets for fungal infections, understanding the regulatory mechanism of such polysaccharides will provide new therapeutic opportunities.

## INTRODUCTION

*Aspergillus fumigatus* is a common opportunistic filamentous fungus that causes invasive infections in immunosuppressed patients (1). Invasive aspergillosis in immune-compromised hosts, e.g., patients suffering from AIDS, receiving organ transplants, undergoing cancer chemotherapy, or treated with corticosteroids, is associated with high mortality (2–5). Despite the emerging increase of individuals susceptible to fungal infections and the rising rates of antifungal drug resistance, the availability of antifungal drugs is still very limited (6, 7). Furthermore, the currently approved clinically used antifungals include echinocandins, triazoles, and amphotericin B, which are relevant to distinct undesired clinical effects (8–12). As a result, there is an urgent need to develop new antifungal strategies and identify novel targets to combat invasive fungal diseases.

Since the cell wall is essential to fungal viability and their absence from human hosts, new drugs disrupting cell wall biosynthesis have gained more attention (13–15). The cell wall of the *A. fumigatus* consists of linear and branched polysaccharides, including chitin, glucans, galactomannan, and galactosaminogalactan (GAG) (16, 17). Despite sharing common substrates and intermediates in their biosynthetic pathways, each cell wall polysaccharide has distinct catalytic and regulatory mechanisms, which makes the cell wall biogenesis of *A. fumigatus* very complex. For instance, two structurally different families containing eight chitinases are responsible for the chitin biosynthesis in *A. fumigatus* (18). Meanwhile β-1,3-glucan is synthesized by a β-1,3-glucan synthase Fks1 (19) and elongated by the β-1,3-glucanosyltransferase Gel family (named Gel1-7), and each Gel is predicted as glycosylphosphatidylinositol (GPI)-anchored protein (16), among which Gel4 is an essential gene (20). The formation of α-1,3-glucan is catalyzed by three paralogous proteins (Ags1/2/3) (21, 22). The biosynthesis of galactomannan requires the coordinated action of galactofuranosyltransferases and mannosyltransferases to assemble short side chains of β-1,3-galactofuranose residues and linear α-mannan backbone (16). GAG is an exopolysaccharide that binds to the cell wall, and a cluster of five genes (*uge3*, *agd3*, *ega3*, *sph3*, and *gtb3*) encode the enzymes required for the biosynthesis of this heteropolysaccharide (23). Antifungal agents, such as caspofungin (CAS) and fosmanogepix, target the β-1,3-glucan synthase Fks1 and the GPI-anchored protein Gwt1 of β-1,3-glucan remodeling to inhibit the growth of pathogenic fungi (24–27), highlighting the importance of the biosynthesis and modification of β-1,3-glucan as antifungal strategies.

The survival of fungal cells is largely dependent on the composition, organization, and function of cell walls, which biosynthesis and remodeling are highly regulated. Multiple transcription factors are reported to play key roles in the transcriptional regulation of pathways implicated in cell wall biosynthesis and remodeling. For example, the calcium-dependent transcription factor CrzA and ZipD could control specific chitin synthase gene expressions in response to different concentrations of caspofungin (28–30). Furthermore, RlmA is a MADS-box transcriptional regulator of cell wall integrity and is important for the proper expression of chitin biosynthesis genes and β-1,3-glucan synthase gene *fks1*, as well as α-1,3-glucan biosynthesis genes *ags1/2/3* (31). Our previous study also revealed that the transcription factor SomA plays an important role in cell wall polysaccharide biosynthesis by directly binding to the conserved motif upstream of GAG biosynthetic genes (*agd3* and *ega3*) and genes involved in cell wall chitin (*chsE* and *chsF*) and β-glucan (*fks1*) biosynthesis (32). Although the transcription factors HapB and ZfpA contribute to cell wall stress response and cell wall integrity, their downstream targets are still unclear (33–35). Hence, little is known about the regulators that govern the expression of the GPI-anchored protein Gels required for cell wall β-1,3-glucan elongation.

MeaB is a basic-region leucine zipper (bZIP) transcription factor that has been identified for the first time as a regulator for nitrogen metabolite repression in *Aspergillus nidulans* (36, 37). In the plant pathogenic fungus *Fusarium oxysporum,* MeaB negatively regulate virulence functions by controlling the expression of virulence-related genes during *F. oxysporum* infection (38). Meanwhile, in *Aspergillus flavus,* there was no evidence of increased virulence of the Δ*meaB* strain; only the *meaB* overexpression strain appears to have decreased virulence (39). The potential role of MeaB in *A. fumigatus* needs to be identified.

SomA and its orthologs play a conserved role in fungal virulence attributes (40–42). The transcriptome sequencing (RNA-seq) and chromatin immunoprecipitation coupled to sequencing (ChIP-Seq) of SomA have been successfully exploited to identify the regulatory mechanisms of fungal virulence-related processes in *A. fumigatus* (32). In this study, we identified a direct downstream target of SomA, MeaB, which possesses several pathogenicity-related characteristics. The loss of *meaB* caused severe defects in the biofilm formation, cell wall integrity, and virulence of *A. fumigatus*. Transcriptional profile analysis revealed that MeaB positively regulates the expression of the GAG biosynthesis and β-1,3-glucanosyltransferase genes *uge3*, *agd3*, and *sph3* and *gel1*, *gel5,* and *gel7*, respectively, as well as genes involved in amino sugar and nucleotide sugar metabolism. Further study demonstrated that the bZIP transcription factor MeaB is a stress response effector and contributes to the proper expression of mitogen-activated protein kinase genes *mpkA* and *mpkC* in the presence of different concentrations of congo red (CR). Thus, we illuminate a mechanism of transcriptional control fungal cell wall polysaccharide biosynthesis and stress response.

## RESULTS

### ChIP-seq and transcriptome analysis identified a number of novel SomA target genes that play a role in biofilm formation and cell wall integrity

**Fig 1:**
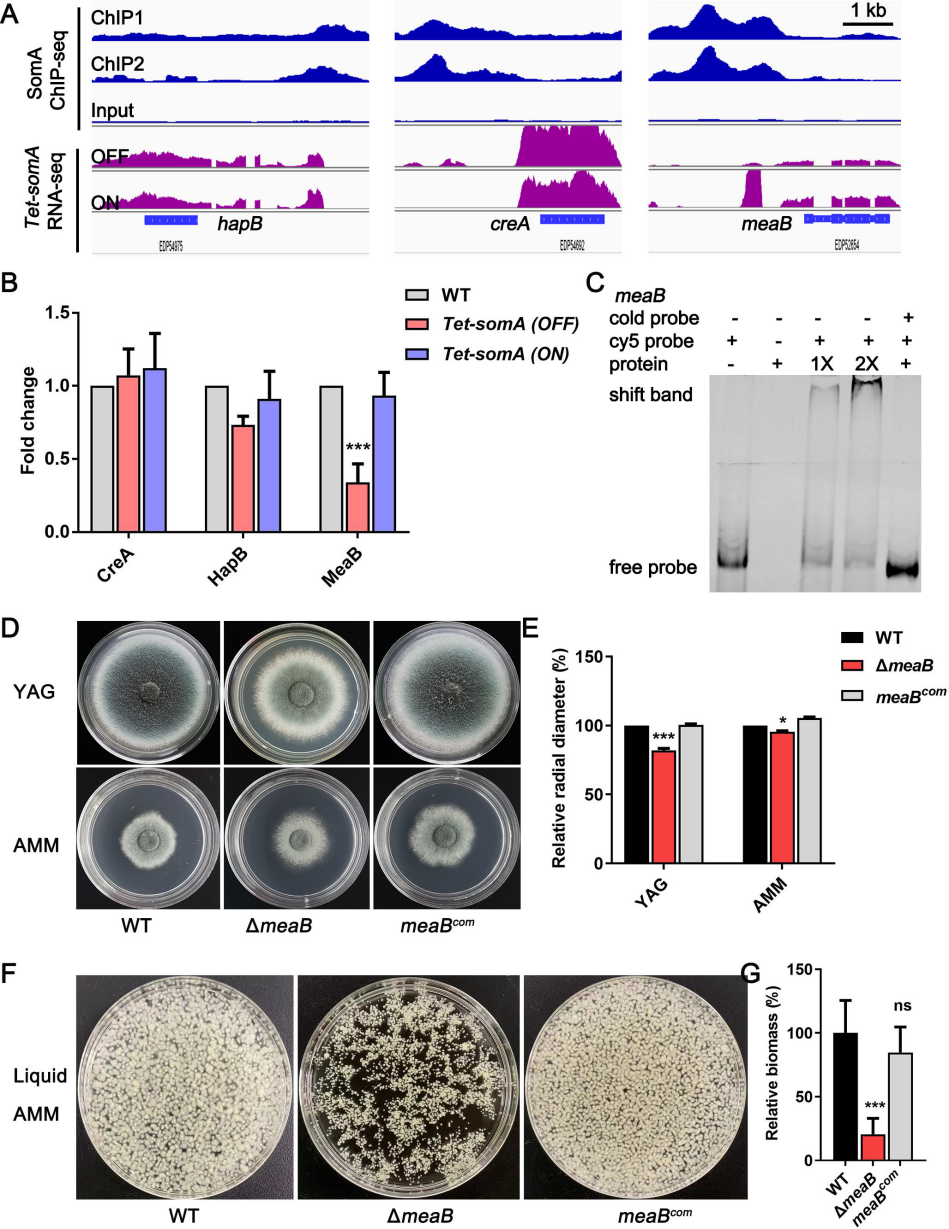
The bZIP transcription factor MeaB is a direct downstream target of SomA. (**A**) Genome browser images depicting the enrichment and transcript levels of SomA on the *hapB*, *creA*, and *meaB* genes based on ChIP-seq (blue) and RNA-seq (purple). ChIP1 and ChIP2 are two independent repetitions. Scale bar, 1 kb. (**B**) RT-qPCR analysis of the relative expression levels of *hapB*, *creA*, and *meaB* in *somA* mutant (*Tet-somA* OFF). The *A. fumigatus* strains were cultured in AMM with (*Tet-somA* ON, induce the expression of SomA) or without doxycycline for 24 h. Gene expression was normalized to the endogenous reference gene *tubA*. The results were performed by three independent biological experiments. (**C**) Electrophoretic mobility shift assay (EMSA) of SomA binding to the promoter fragments of *meaB*. The specificity of EMSA binding was confirmed by adding specific cold probe competitors (50-fold unlabeled probe). (**D**) Phenotypes of the WT and Δ*meaB* and *meaB^com^* strains grown on solid rich medium (YG) or minimal medium (AMM) at 37°C for 48 h. (**E**) Quantitative analysis of relative colony diameter of the indicated strains. The data are presented as the percentages of the colony diameter of the WT strain, and the results are the means of three repetitions ± standard deviation (SD). (**F and G**) Phenotypes and quantifications of the WT and Δ*meaB* and *meaB^com^* strains grown in liquid minimal medium at 37°C for 48 h. ^*^P < 0.05; ^***^P < 0.001. ns, not significant.

Previous studies confirmed that SomA is a key transcription factor of *A. fumigatus*, which controls fungal development, biofilm formation, cell wall stress response, and virulence (32, 40). To investigate the transcriptional network connects to these crucial pathways, we identified and functionally characterized a number of transcription factors downstream of SomA based on our previous ChIP-seq and RNA-seq data (32). Among the 426 direct targets bound by SomA in ChIP-seq analyses, there were 29 genes belonging to different family transcription factors (see Table S1). The 29 single mutants from a library of *A. fumigatus* TF null mutants (43) were selected to evaluate whether they play a critical role in biofilm formation and cell wall integrity. As shown in Fig. S1, the biofilm biomass of *hapB*, *creA*, and *meaB* null mutants was significantly decreased compared to that of the *A. fumigatus* wild type (WT). Three of the deletion mutants (Δ*hapB*, Δ*creA*, and Δ*meaB*) were also hypersensitive to the cell wall-perturbing agent congo red (Fig. S1), suggesting the potential roles in cell wall integrity. Furthermore, the evaluation of the ChIP-seq data suggested that SomA was predominantly occupied in the promoter region of these genes (Fig. 1A). The transcript levels of three indicated transcription factors based on RNA-seq demonstrated a reduced abundance of *meaB* mRNA, but not *creA* or *hapB*, in the *somA* mutant strain, suggesting a direct regulation of transcription factor MeaB by SomA (Fig. 1A). Consistent with these findings, reverse transcription quantitative polymerase chain reaction (RT-qPCR) confirmed that the expression of *meaB* was significantly decreased in the *somA* mutant [*Tet-somA* strain (OFF)] grown in the AMM compared with that in the WT strain (Fig. 1B). Consequently, the electrophoretic mobility shift assay further confirmed the *in vitro* binding of SomA to the conserved “GTACTCCGTAC” motif-containing promoter fragments of *meaB* (Fig. 1C). Considering the regulatory mechanism of *A. fumigatus,* CreA in biofilm formation and cell wall integrity has been elucidated (44, 45). Our results of HapB in cell wall integrity and biofilm formation (Fig. S1 and S2) were also consistent with the previous findings that it is a cell wall stress response factor (34, 35) and that it is also a potential regulator of GAG polysaccharide biosynthesis genes based on the *hapB* mutant RNA-seq data (46), respectively. In the present study, we focus on the regulatory mechanism of MeaB on these fungal pathogenicity-related pathways.

To explore the biological functions of *A. fumigatus* MeaB, a Δ*meaB* null mutant and complemented (*meaB^com^*) strains were generated. The complementation strain *meaB^com^* was constructed by the ectopic insertion of the target gene into the genome of the Δ*meaB* background strain. As shown in Fig. 1D and E, the Δ*meaB* mutant strain showed markedly reduced radial growth on solid rich medium (YG) and minimal medium (AMM) compared to the WT and complemented strains. Moreover, consistent with the decreased radial growth on solid medium, the Δ*meaB* mutant exhibited a significantly reduced biomass when submerged into liquid AMM for 24 h (Fig. 1F and G). Collectively, the above results suggest that the bZIP transcription factor MeaB is a direct downstream target of SomA, which regulates *A. fumigatus* hyphal growth.

### MeaB deficiency causes reduced GAG production, adhesion, and attenuated virulence in *A. fumigatus*

**Fig 2:**
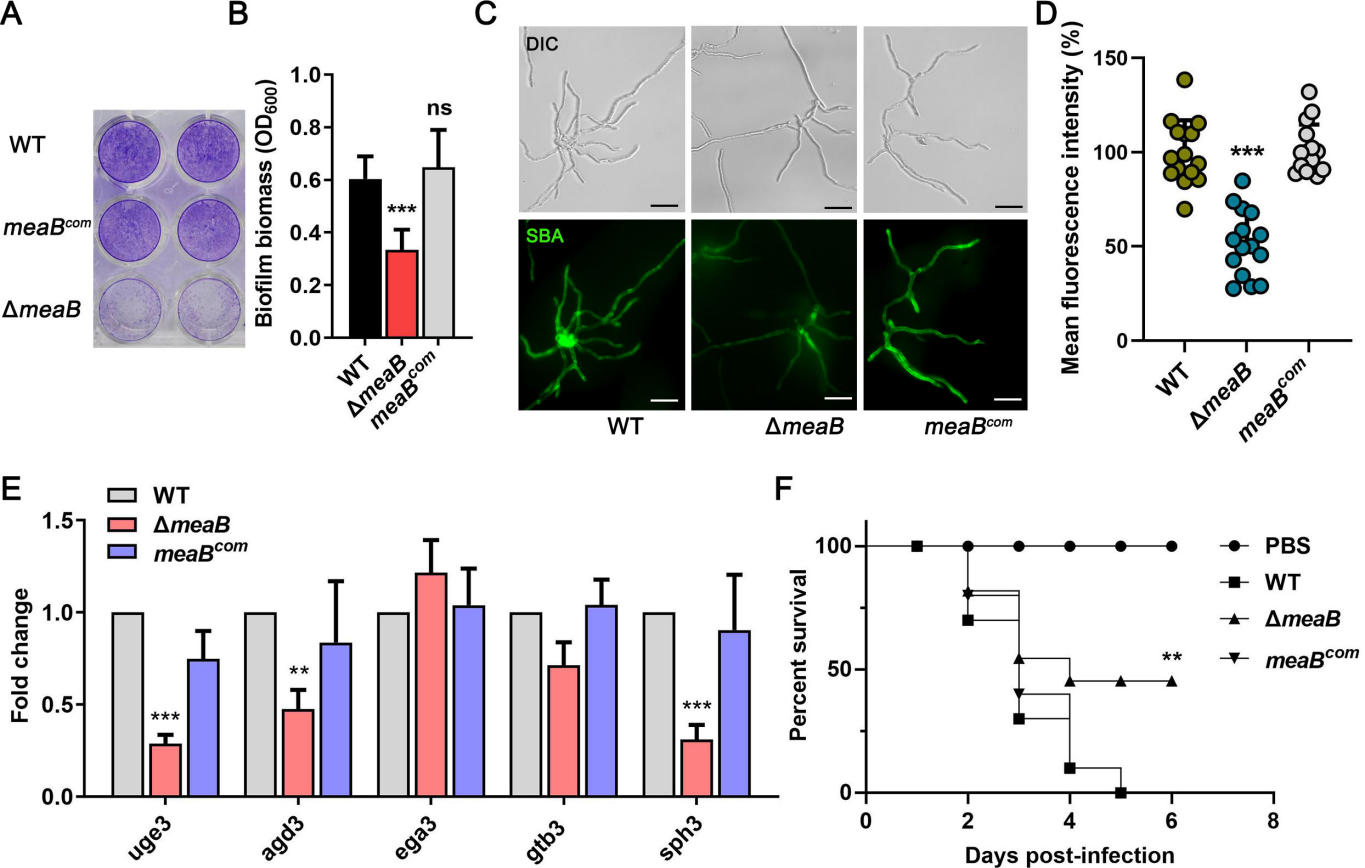
MeaB is involved in biofilm formation and virulence of *A. fumigatus*. (**A**) Formation of adherent biofilms by the WT and Δ*meaB* and *meaB^com^* strains after 24 h of growth on polystyrene plate surfaces in AMM. (**B**) Biofilms were visualized by staining with crystal violet and quantified by determining the absorbance at 600 nm. (**C**) Representative images of hyphae of the WT and Δ*meaB* and *meaB^com^* strains stained with SBA-FITC after growth in RPMI 1640. Scale bar, 10 µm. DIC, differential interference contrast. (**D**) Quantification of the mean fluorescence intensity (MFI) of *A. fumigatus* hyphae grown under the same conditions as the ones described above for panel (C). Data are presented as the percentages of the MFI of the WT strain, and the means ± SD are from three independent biological samples, each with five hyphal sections measured (^***^P < 0.001). (**E**) RT-qPCR analysis of the relative expression levels of the GAG cluster genes in the WT and Δ*meaB* and *meaB^com^* strains after 24 h of growth in MM. Gene expression was normalized to the endogenous reference gene *tubA*. Results represent data from three independent biological experiments. (**F**) Survival curves for *G. mellonella* larvae infected with the WT and Δ*meaB* and *meaB^com^* strains; PBS-injected larvae were used as a negative control. Statistical analysis between groups used the log-rank test (^**^P < 0.01).

Given the crucial role of SomA in the adhesion of *A. fumigatus*, we believed that the downstream target of SomA, *meaB,* may play a role in mediating biofilm formation. The formation of adherent biofilms was visualized by staining with crystal violet. As shown in Fig. 2A and B, in comparison to the parental and complemented strains, the Δ*meaB* mutant was markedly impaired in the formation of adherent biofilms on plastic, representing half of the total amount of biofilm biomass produced by the parental strain in liquid AMM. Since GAG functions as the dominant adhesin of *A. fumigatus* and mediates biofilm formation, we hypothesized that the reduced augmentation of biofilm production might reflect a decrease in GAG production. Consistent with this hypothesis, the immunofluorescence staining of GAG by fluorescein-tagged soybean agglutinin lectin (SBA-FITC) revealed that the distribution of GAG production on the hyphal surface of Δ*meaB* mutant was significantly decreased (Fig. 2C and D). RT-qPCR analysis confirmed that the expressions of GAG biosynthesis genes *uge3*, *agd3,* and *sph3* were dependent on MeaB, suggesting that transcription factor MeaB governs the biofilm formation by regulating the expression of GAG biosynthesis-related genes (Fig. 2E). GAG is an important virulence factor in *A. fumigatus*, and multiple studies have found that certain regulatory factors that govern GAG biosynthesis are required for full virulence of *A. fumigatus* (32, 40, 47–50). To determine if MeaB contributes to fungal pathogenesis, the effects of the Δ*meaB* mutant were assessed in a *Galleria mellonella* infection model. Larvae were infected with 1 × 10^6^ *A. fumigatus* conidia per larva of the indicated strains, and survival was monitored at 37°C. Survival analysis revealed that loss of *meaB* resulted in a significantly improved survival rate (*P* < 0.01) of larvae compared to the WT strain 6 days post-infection (Fig. 2F). In summary, these data show that MeaB regulates GAG biosynthesis gene expression and contributes to full virulence of *A. fumigatus*.

### MeaB is involved in the maintenance of cell wall integrity

**Fig 3:**
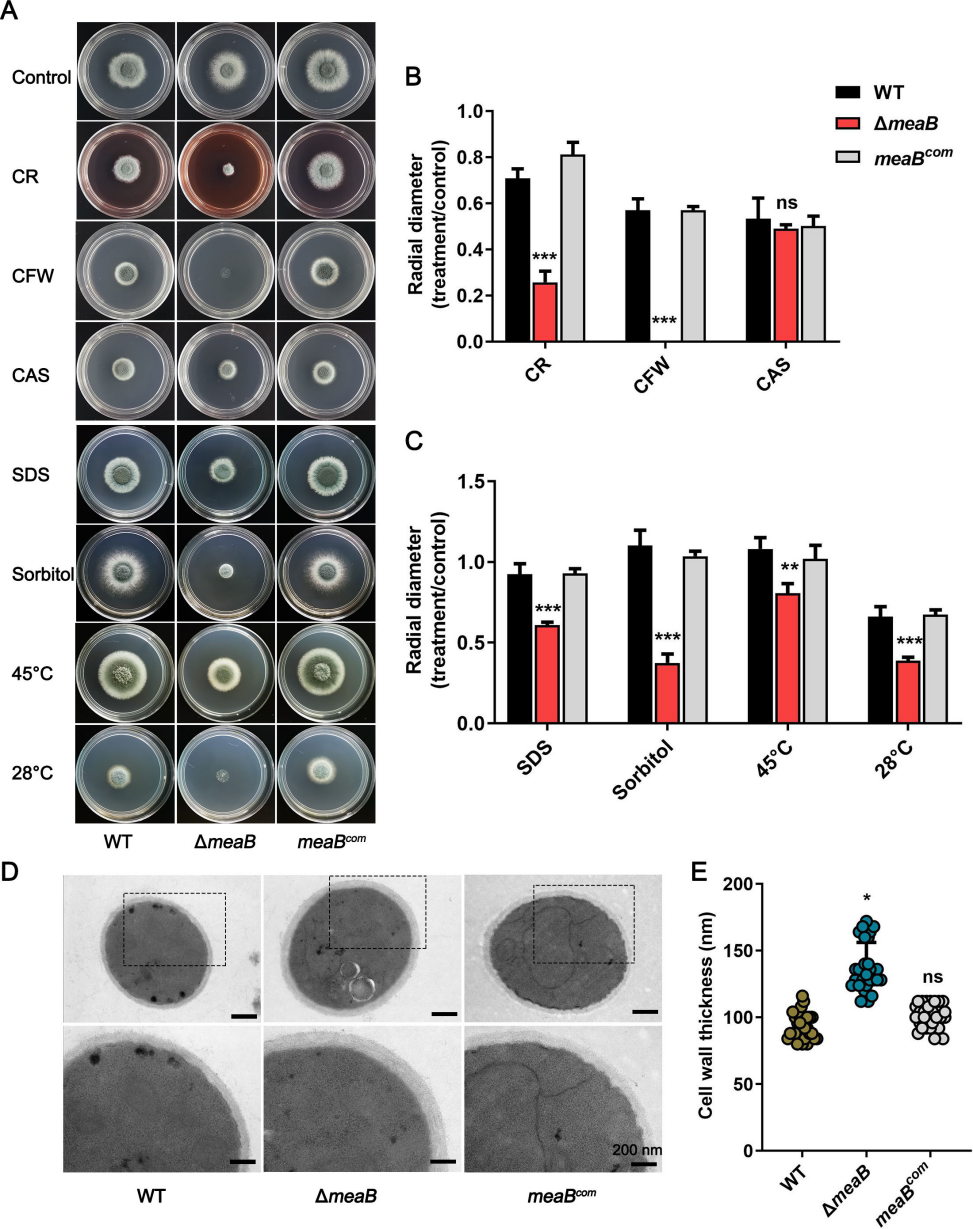
Loss of *meaB* causes increased hypersensitivity to cell wall stress and cell wall thickness of hyphal germlings. (**A**) Phenotypes of the WT and Δ*meaB* and *meaB^com^* strains cultured on AMM at 37°C, 45°C, or 28°C or AMM supplemented with CR, CFW, CAS, SDS, or 1.2 M sorbitol at 37°C. Colony morphology was imaged after 48 h. (**B and C**) Quantitative analysis of relative colony diameter of the WT and Δ*meaB* and *meaB^com^* strains. The data are presented as the ratio of treatment to control groups of the indicated strain, and the results are the means of three repetitions ± SD. (**D**) Representative TEM images of hyphae of the WT and Δ*meaB* and *meaB^com^* strains cultured in MM. Scale bar, 200 nm. (**E**) Quantification of the mean cell wall thickness of the WT and Δ*meaB* and *meaB^com^* strains as in panel (A). Data are presented as the means ± SD of three biological samples, with 10 sections measured for each. ^*^P < 0.05; ^***^P < 0.001.

Next, we analyzed the growth of the Δ*meaB* mutant strain under different cell wall perturbing conditions. As shown in Fig. 3A through C, the Δ*meaB* mutant strain presented increased sensitivity to chitin-binding agents on AMM plates, such as CR and calcofluor white (CFW), but did not show any sensitivity to the β-glucan synthase inhibitor CAS. Furthermore, we also observed that loss of *meaB* resulted in a hypersensitivity to the detergent sodium dodecyl sulfate (SDS) and high osmotic stress conditions (1.2 M sorbitol). Interestingly, lack of *meaB* not only causes a high temperature (45°C) sensitivity phenotype but also exhibits increased growth defects under low temperature (28°C) conditions (Fig. 3B and C), indicating that the cell membrane of *A. fumigatus* may also be affected by the *meaB* mutant. Besides, it is reported that the bZIP transcription factor MeaB is a fungal nitrogen metabolism regulator, and the growth phenotypes of such mutant will be different on distinct nitrogen source plates. Therefore, we performed the cell wall stress assay on another minimal medium with nitrate as the sole nitrogen source (NMM). In agreement, the *A. fumigatus* Δ*meaB* mutant strain showed significant growth defects on NMM supplemented with CR and CFW, but not on the CAS plate (Fig. S3). Given that the cell wall architecture is crucial for fungal stress response, we performed transmission electron microscopy assays to observe the influence of MeaB on *A. fumigatus* hyphal germlings. The thickness of the cell wall in the Δ*meaB* mutant strain was found to be 1.4-fold (*P* < 0.05) thicker than that in the WT or complemented strains (Fig. 3D and E), indicating that MeaB plays a potential role in cell wall architecture maintenance.

### MeaB regulates fungal carbohydrate metabolism and cell wall-related polysaccharide biosynthesis

**Fig 4:**
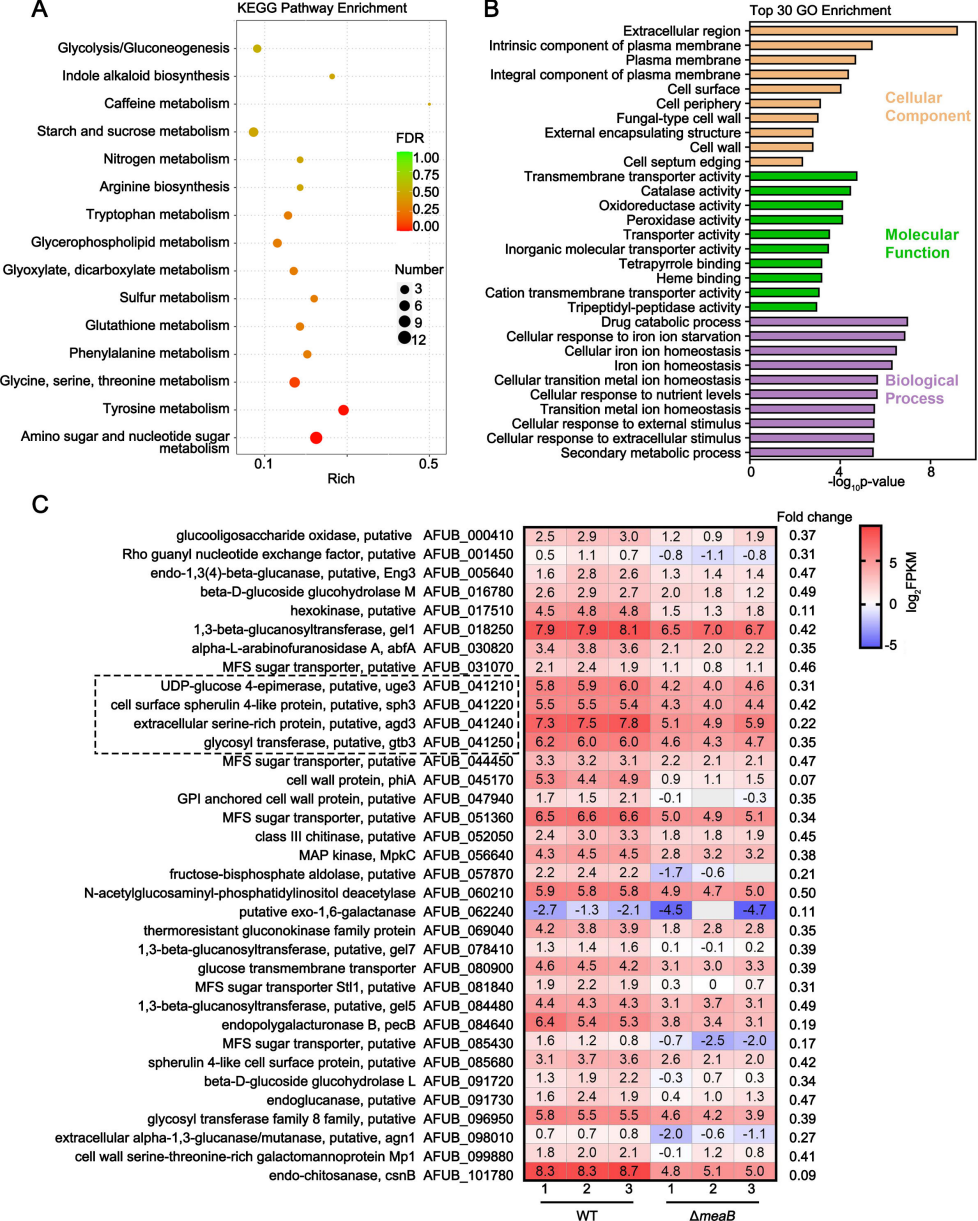
MeaB regulates fungal cell wall biogenesis. (**A**) KEGG pathway enrichment of differentially expressed genes in the Δ*meaB* mutant strain compared to the WT. False discovery rate values and gene numbers are represented using a gradient of color and bubble size, respectively. (**B**) Bar charts show the GO enrichment analysis of the DEGs in the *meaB* mutant. The 30 most significantly enriched GO terms of biological process, cellular components, and molecular function were selected as representations. (**C**) The heat map shows the selected genes (FC >2 and *P* < 0.05) putatively involved in the cell wall polysaccharide metabolism pathway. Blank gray cells indicate missing FPKM values, FPKM, fragments per kilobase per million.

To further elucidate the regulatory mechanism underlying MeaB-mediated cell wall integrity, we measured the global gene expression changes in the Δ*meaB* mutant strain compared to the WT strain cultured in liquid AMM at 37°C for 24 h by RNA-seq. RNA-seq identified 819 (333 up- and 486 downregulated) (Fig. S4) differentially expressed genes (DEGs) (fold change of >2; *P* < 0.05) in the Δ*meaB* mutant strain (Table S2). The Kyoto Encyclopedia of Genes and Genomes (KEGG) pathway enrichment analysis of the DEGs revealed that the significantly enriched pathways were glycine, serine, threonine metabolism, and tyrosine metabolism, as well as amino sugar and nucleotide sugar metabolism (Fig. 4A), a key pathway providing precursors for the biosynthesis of cell wall and GAG polysaccharides. To identify the potential physiological roles of the MeaB-dependent genes in specific fungal processes, we subjected these DEGs to Gene Ontology (GO) analysis. Consistent with our KEGG analysis, the significantly enriched genes with functions in the cellular component were associated with the extracellular region, plasma membrane, and fungal-type cell wall (Fig. 4B). Besides, these DEGs were significantly enriched in genes with functions in diverse processes, including trans-membrane transport processes, iron ion homeostasis, and cellular response external stimulus processes (Fig. 4B). Of note, among the DEGs dependent on MeaB, we observed that the expression of genes in cell wall polysaccharide metabolism pathways was significantly downregulated, including the beta-glucanase Eng3, the 1,3-beta-glucanosyltransferases Gel1/5/7, and the hexokinase AFUB_017510, as well as a kind of major facilitator superfamily sugar transporter (Fig. 4C). Most importantly, we found that GAG polysaccharide biosynthesis cluster genes *uge3*, *agd3*, *gtb3*, and *sph3* were significantly downregulated (fold change >2, *P* < 0.05) (Fig. 4C), which is consistent with the above RT-qPCR analysis. Collectively, these results suggest that MeaB is involved in fungal carbohydrate metabolism and cell wall-related polysaccharide biosynthesis.

### MeaB regulates the expression of cell wall glucanosyltransferase genes *gel1*, *gel5*, and *gel7* and the mitogen-activated protein kinase genes *mpkA* and *mpkC*

**Fig 5:**
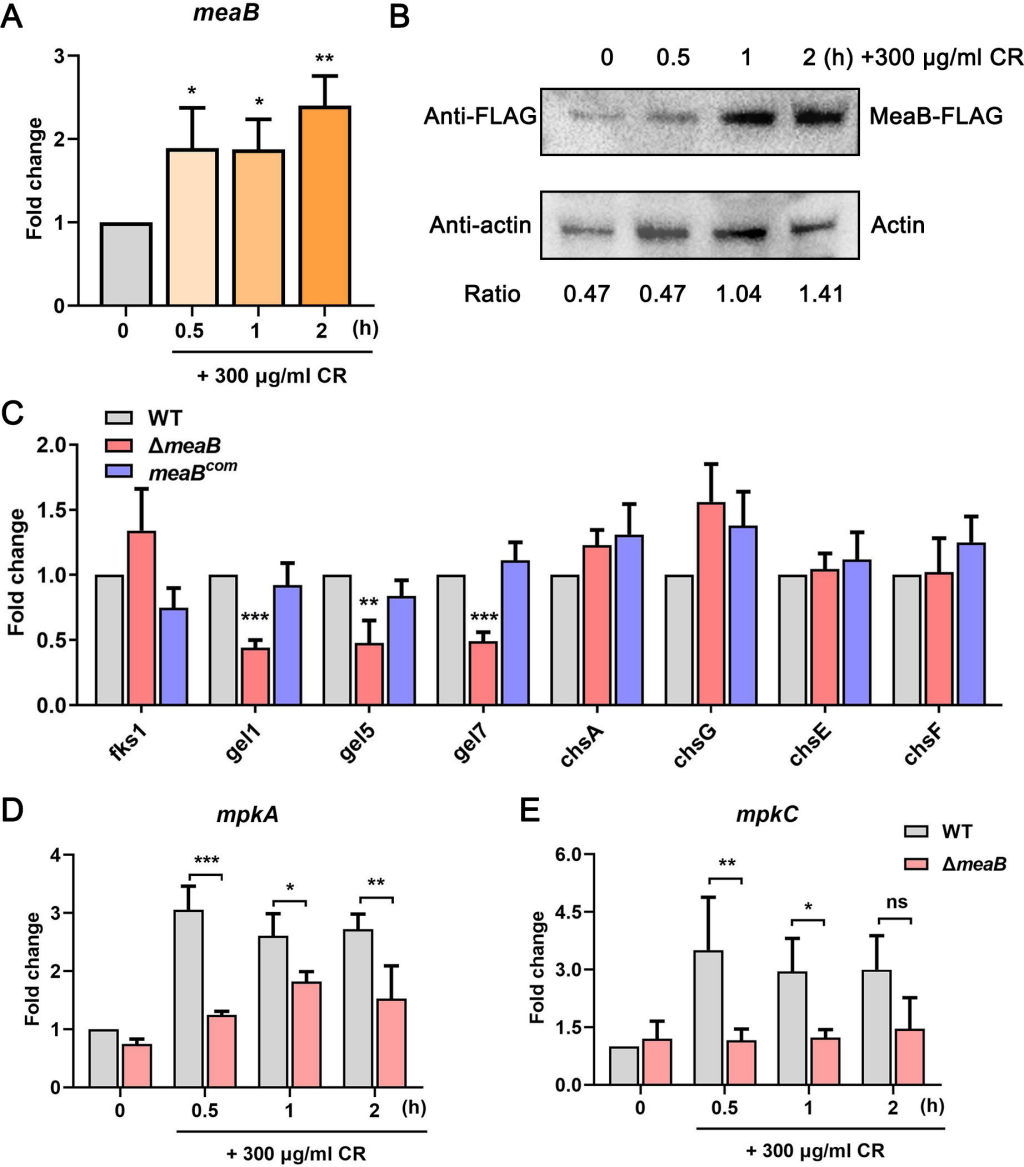
The stress response effector MeaB regulates the expression of cell wall glucanosyltransferases and mitogen-activated protein kinases. (**A and B**) RT-qPCR and western blotting analysis of the relative expression levels of *meaB* in the WT in the presence of 300 µg/mL CR for 0.5, 1, or 2 h. Gene expression was normalized to the endogenous reference gene *tubA*. Quantified western blot signal intensity was performed by ImageJ software. (**C**) RT-qPCR analysis of the relative expression levels of cell wall biosynthesis-related genes in the WT and Δ*meaB* and *meaB^com^* strains after 24 h of growth in MM. (D and E) RT-qPCR analysis of the relative expression levels of *mpkA and mpkC* in the WT and Δ*meaB* mutant in the presence of 300 µg/mL CR for 0.5, 1, or 2 h. Results represent data from three independent biological experiments. ^*^P < 0.05; ^**^P < 0.01; ^***^P < 0.001.

The RNA-seq GO analysis indicated that MeaB is a potential stress response effector; to do so, we monitored the mRNA levels of *meaB* in the WT strain when grown in liquid AMM for 24 h and after the addition of 300 µg/mL CR for 0.5, 1, and 2 h. As expected, the expression of *meaB* was significantly induced by 300 µg/mL CR stress (Fig. 5A). Next, we sought to determine the expression patterns of MeaB at the protein level. We labeled MeaB with a FLAG tag at the C-terminus and expressed the fusion protein under the control of MeaB native promoter in the parental strain. Plate assays confirmed that MeaB-FLAG function well and do not change the sensitivity of *A. fumigatus* to CR or CFW (Fig. S5). In agreement with RT-qPCR results, western blotting analysis showed that the protein expression of MeaB was improved by 300 µg/mL CR treatment (Fig. 5B). Therefore, we speculate that the transcription factor MeaB regulates the fungal cell wall integrity by increasing its expression level in response to cell wall stress. To further explain the regulatory mechanism of MeaB on cell wall integrity, RT-qPCR was performed to detect the expressions of cell wall chitin- and glucan-related synthases. As shown in Fig. 5C, the expression levels of cell wall glucanosyltransferases *gel1*, *gel5,* and *gel7* were significantly downregulated in the Δ*meaB* mutant strain compared to the WT strain cultured in liquid AMM for 24 h, which also confirms our RNA-seq data. However, we did not observe any difference in the expressions of cell wall β-glucan synthases *fks1* and representing two classes of chitinases *chsA*/*G* and *chsE*/*F* between the Δ*meaB* mutant and WT strains, under the current experimental conditions (Fig. 5C). In addition, we also measured the relative expression levels of these cell wall biosynthesis related-genes under the CR stress conditions. Consistently, there was no significant difference in the relative expression levels of these genes between the *meaB* mutant and WT strain under distinct stress conditions (Fig. S6).

As we know, MpkA and MpkC are two important regulators of fungal cell wall integrity and stress response. MpkA is a central regulator of the cell wall integrity pathway, and its role was associated with the response to cell wall disturbing compounds, while the function of MpkC was related to signaling required for carbon source utilization and osmo-stress response (51–54). As expected, the expression of the *mpkA* gene was dramatically induced by CR treatment in the WT strain (Fig. 5D). Strikingly, the CR-mediated increase in the expression of *mpkA* gene that appeared in the WT strain was repressed in the Δ*meaB* mutant strain, suggesting the stress response of MpkA partial dependence on MeaB (Fig. 5D). Similar to *mpkA*, we found that the expression of the *mpkC* gene was significantly induced for about threefold higher by CR treatment for 0.5, 1, and 2 h, and the deletion of *meaB* almost abolished the CR stress response of *mpkC* (Fig. 5E). Collectively, these results suggest that stress response effector MeaB regulates cell wall integrity by governing the expression of cell wall glucanosyltransferase genes *gel1*, *gel5*, and *gel7* and the mitogen-activated protein kinase genes *mpkA* and *mpkC.*

## DISCUSSION

The fungal cell wall is a dynamic organelle important for pathogen survival, and its composition and structural organization are frequently reshuffled depending on the environmental conditions (16). While the remodeling of the fungal cell wall structure is a complex and tightly regulated process, the underlying mechanism is still not fully understood. It has been shown that the transcription factor SomA regulates *A. fumigatus* cell wall architecture and composition by governing the expression of cell wall chitin and β-glucan polysaccharide biosynthesis genes (32). In the present study, we identify and functionally characterize a bZIP transcription factor MeaB, which is a direct transcriptional target of SomA. We show that the deletion of *meaB* leads to severe defects in biofilm formation and attenuates virulence in a *Galleria mellonella* infection model, as well as hypersensitivity to cell wall stress agents, which is similar to the critical functions of SomA in *A. fumigatus*. However, different from SomA, the major novel findings from the study of MeaB are that MeaB is a stress response effector and contributes to the proper expression of both *mpkA* and *mpkC* and that MeaB regulates the expression of cell wall glucanosyltransferase genes *gel1*, *gel5*, and *gel7*, but not chitinase genes or β-glucan synthase *fksA*. As we know, the GEL family proteins play key roles in splitting and remodeling the β-glucan molecule, resulting in the elongation of the glucan chain (16). In addition, *gel1* and *gel7* also contribute to the cell wall composition and integrity of *A. fumigatus* (55, 56), which is consistent with the present findings of MeaB. Furthermore, the deletion of *meaB* altered the cell wall architecture of *A. fumigatus*, which may be one of the reasons for the sensitivity of Δ*meaB* mutant to CR or CFW. As for MpkA, the protein is a canonical mitogen-activated protein kinase involved in fungal cell wall integrity (51, 57). The reduced expression of *mpkA* in Δ*meaB* mutant may be one of the reasons for *A. fumigatus* decreased resistance to cell wall-perturbing agents. However, further study is needed to determine whether the phosphorylation level of MpkA will be affected by the Δ*meaB* mutant. Likewise, we observe that MeaB also seems to play a role in osmotic stress response in the current assay, as the mutant strain exhibited hypersensitivity to 1.2 M sorbitol stress. In agreement with this finding, the RNA-seq and RT-qPCR analysis reveal a regulatory role of MeaB on MpkC, a key osmo-stress response kinase. MpkC also plays an important role in carbon source metabolism and cell wall stress response (53, 54), which might also account for the increased susceptibility of *A. fumigatus* Δ*meaB* to the treatment with CR and CFW, underscoring an important role of MeaB in cell wall integrity.

Notably, cell wall organization and GAG production directly impact virulence and host immune recognition in *A. fumigatus* (58–61). Based on the aforementioned results, we demonstrate that MeaB contributes to full virulence in a *Galleria mellonella* infection model of *A. fumigatus.* An association with virulence traits of MeaB has been previously reported in other fungi. In the *F. oxysporum* study, loss of *meaB* resulted in increased virulence in ammonium-supplemented pathogenicity assays (38, 62). Whereas there was no evidence of increased virulence of the *A. flavus* Δ*meaB* mutant strain, interestingly, the *meaB* overexpression strain was reduced in virulence (39). Thus, the virulence-related mechanisms driven by these fungi are largely dependent on the distinct transcriptional regulation of pathways implicated in virulence. For example, MeaB normally inhibits the Ste12 activation of a MAPK signaling pathway in *F. oxysporum* (62)*,* and loss of this inhibition could explain the enhanced virulence of the Δ*meaB* mutant strain. In *A. flavus,* the authors revealed an opposite regulation of *ste12* by *meaB* (39), suggesting that the contribution of *meaB* on virulence in *A. flavus* does not act through the same signaling pathways as *F. oxysporum*. The involvement of cell wall integrity, biofilm formation, and stress response in fungal pathogenicity and virulence has already been validated. Therefore, the virulence characteristics of *A. fumigatus* Δ*meaB* may originate from the divergence control mechanisms for these distinct networks.

The cell wall and extracellular polysaccharides, including glucans, chitin, galactomannan, and GAG, share multiple common substrates and intermediates in the biosynthesis of these polymers. Many proteins playing a certain role in cell wall biogenesis are also involved in the regulation of biofilm GAG biosynthesis. Apart from the aforementioned transcription factors SomA and MeaB, a salient case is that of CreA; the carbon catabolite repressor not only regulated cell wall homeostasis (44) but was also closely associated with galactosaminogalactan-mediated biofilm formation (45). Here, our data indicate that loss of the CCAAT-binding transcriptional regulator, HapB, causes severe defects in congo red resistance and GAG detection. These results are consistent with the observation that *hapB* deletion results in an increased susceptibility to cell wall stress agent caspofungin and decreased expression of GAG biosynthesis-related genes based on the previous RNA-seq data (34, 35, 46). Similarly, the MADS-Box transcription factor RlmA and the histone acetyltransferase GcnE also seem to be involved in these two pathways (31, 63). In summary, these results reveal that the biosynthesis of these distinct cell wall polysaccharides is a complex and interconnected process. The evidence can also be gained from another perspective; the transcription factors SomA (an ortholog in *Saccharomyces cerevisiae* is Flo8), HapB, and CreA are protein kinase A (PKA)-dependent targets (34, 64, 65), while the transcription factor RlmA is a down effector of MpkA (31, 66). It is worth noting that both PKA and MpkA signal pathways play critical roles in fungal cell wall integrity and that both of the kinase modules contribute to fungal adhesion and biofilm formation (67–71), although biofilm-contained GAG has not yet been found in yeast. In summary, the present study reveals the regulatory network of transcription factors on polysaccharide synthesis in distinct cell wall components.

In conclusion, this study functionally describes a transcription factor MeaB that regulates cell wall integrity and stress response, as well as contributes virulence of *A. fumigatus*. This reinforces our understanding of the fungal cell wall organization and their regulatory mechanisms in the opportunistic fungal pathogen and provides insights into the fungal virulence mechanisms that may aid in the discovery of novel antifungal drug targets (72).

## MATERIALS AND METHODS

### Strains and growth conditions

All strains used in this study are listed in Table S3 in the supplemental material. *A. fumigatus* strains were typically grown at 37°C in rich medium (YG; 2% glucose, trace elements, and 0.5% yeast extract), minimal medium containing 1% glucose as carbon sources, 10 mM ammonium tartrate (AMM) or 70 mM sodium nitrate as nitrogen sources (NMM), trace elements, and pH 6.5, and RPMI 1640 (Sigma-Aldrich, USA). Solid MM and YG were the same as those described above except that 2% agar was added. The *A. fumigatus* A1160::pyrG was used as a wild-type control strain (73).

### Genetic modifications

All primers used in this study are listed in Table S4 in the supplemental material. The Δ*meaB* null mutant strain was generated using a *pyr4* selectable marker strategy, and the fusion PCR method was performed to construct the *meaB* knockout cassette as previously described (74). Briefly, approximately 1.2 kb of the upstream and downstream flanking DNA segments of the *meaB* open reading frame (ORF) was amplified with the primer pairs MeaB-P1/-P3 and MeaB-P4/-P6, respectively. The *pyr4* gene was amplified from plasmid pAL5 with the primer pair Pyr4-F and -R. The resulting PCR products were used as the templates to generate the *meaB* knockout cassette with the primers MeaB-P2 and -P5. The fusion product was cloned into the pEASY-Blunt Zero vector (TransGen Biotech) and transformed into the 1160 recipient strain. Transformants were grown on AMM and verified by diagnostic PCR.

The complemented strain *meaB^com^* was generated by the ectopic insertion of the target gene into the genome of the Δ*meaB* background strain. The full-length ORF and native promoter of *meaB* were amplified from *A. fumigatus* 1160 genomic DNA using the primer pair meaB^com^ -F and -R. The resulting fragment was cloned into the hygromycin B resistance gene *hph*-contained plasmid pAN7-1 and then transformed into the *meaB* knockout strain, and the transformants were selected on the YG medium supplemented with 200 µg/mL hygromycin B (Sangon Biotech).

The FLAG-tagged MeaB strain was constructed using a similar fusion PCR strategy as described above. Three DNA fragments of *flag-pyr4* upstream and downstream flanking sequences of the *meaB* termination codon were amplified with the primer pairs FlAG-SF/Pyr4-R, MeaBflag-P1/-P3, and MeaBflag-P4/-P6, respectively. The resulting PCR products were used as templates to generate *meaB-flag-pyr4* cassette using the primers MeaBflag-P2 and P5, followed by sequencing, and then transformed into the 1160 recipient strain. Transformants were grown on AMM and verified by diagnostic PCR and western blotting.

### Plate assays

To monitor the sensitivity of *A. fumigatus* strains to various cell wall-perturbing agents, 2 µL of 1 × 10^7^ conidia/mL spore suspension was spotted onto minimal medium agar plates that were supplemented with 30 µg/mL CR, 50 µg/mL CFW, 1 µg/mL CAS, 25 µg/mL SDS, or 1.2 M sorbitol. The plates were cultured at 37°C, and images were taken at 48 h.

### Biofilm formation assay

*A. fumigatus* biofilm visualization and quantification were performed as previously described (50), with minor modifications. Briefly, 1 mL of AMM per well containing 2 × 10^5^ *A. fumigatus* conidia was cultured in 24-well polystyrene plates at 37°C for 22 h. Then, the adherent biofilms were washed twice with 500 µL of distilled water and dried at room temperature. Adherent biofilms were stained with 300 µL of 0.1% (wt/vol) crystal violet for 10 min at room temperature. After removing the excess crystal violet solution, the stained biofilms were washed twice with 500 µL of distilled water. The stained adherent biofilms were extracted by adding 1 mL of ethanol. Biofilm biomass was determined by measuring the absorbance of the destained solution at 600 nm.

### Galactosaminogalactan characterization

The hypha surface galactosaminogalactan polysaccharide was charactered by immunofluorescence staining using GAG-specific fluorescein-labeled soybean agglutinin (SBA-FITC) (Vector Labs, USA) (23). In brief, the *A. fumigatus* strains were cultivated on coverslips in RPMI 1640 (Sigma-Aldrich) for 8 to 10 h, subsequently washed with phosphate-buffered saline (PBS), and stained with SBA-FITC for 2.5 h in the dark. After fixation with paraformaldehyde, samples were washed with PBS and microscopically imaged (Zeiss, Germany).

### RT-qPCR and RNA-seq

Fresh *A. fumigatus* conidia were cultured in liquid AMM in a rotary shaker at 200 rpm at 37°C for 48 h. For RT-qPCR analysis, total RNA was isolated using a spin column total RNA purification kit (Sangon Biotech) according to the manufacturer’s instructions. gDNA digestion and cDNA synthesis used the HiScriptII Q RT SuperMix for qRCR kit (Vazyme). The quantitative PCR was carried out using the AceQ qPCR SYBR green master mix kit (Vazyme) on an ABI one-step fast thermocycler (Applied Biosystems). Three independent biological replicates were used, and the gene expression levels were calculated using the 2^−ΔΔ^*^CT^* method (75) and normalized to *A. fumigatus* tubulin. Except for the supplementation with 300 µg/mL CR for 0.5, 1, or 2 h after 24 h of growth of *A. fumigatus*, the stress response assay was the same as described above. For RNA-seq analysis, all sample purification, library construction, and sequencing were performed by Shanghai Personal Biotechnology (China) based on the Illumina sequencing platform. The threshold value of differentially expressed genes was a fold change of >2 and a *P*-value of <0.05. All the samples were evaluated using three biological repetitions.

### Western blotting analysis

The *A. fumigatus* conidia were cultured in liquid AMM in a rotary shaker at 200 rpm at 37°C for 48 h, followed by being exposed to 300 µg/mL CR for 0.5, 1, or 2 h. The mycelia were collected and ground in liquid nitrogen with a mortar and pestle. Total protein isolation was carried out in alkaline lysis buffer (0.2 M NaOH and 0.2% β-mercaptoethanol) as previously described (76). The lysate samples were loaded on 10% SDS-PAGE gel and blotted on a polyvinylidene difluoride membrane (Millipore) and then hybridized with anti-FLAG (Sigma-Aldrich, F3165) and anti-β-actin (ABclonal, AC026). The secondary antibodies were peroxidase-labeled goat anti-mouse (Proteintech, SA00001-1) and goat anti-rabbit (Proteintech, SA00001-2) and detected by an enhanced ECL luminescence detection kit (Vazyme) and then visualized by the imaging system (Bio-Rad). The band intensity was calculated using ImageJ software.

### SomA protein expression, purification, and electrophoretic mobility shift assay

Recombinant SomA prokaryotic expression, purification, and *cy5* probe labeling were carried out as described previously (32). EMSA of SomA was performed in a 20-µL reaction mixture containing 1 µg recombinant protein (1×), 50 ng probe DNA, and EMSA buffer (40 mM Tris-HCl, pH 8.0, 50 mM NaCl, 10 mM MgCl_2_, 5% glycerol). The resulting mixes were pre-incubated at 37°C for 30 min and then separated on a 5% polyacrylamide gel in 0.5 Tris-borate EDTA buffer and subsequently imaged.

### Transmission electron microscopy analysis of the cell wall

The thickness of the cell wall of hyphal germlings was determined by TEM, as previously described (29, 32, 77). Briefly, fresh *A. fumigatus* conidia were cultured in liquid AMM at 37°C for 12 h and then fixed overnight in 0.1 M sodium phosphate buffer (pH 7.4) containing 2.5% glutaraldehyde at 4°C. The samples were sequentially embedded in 1% (wt/vol) agar, fixed in 1% OsO_4_, dehydrated in ethanol, embedded in epoxy resin monomer (SPI), and then sliced using an ultra-thin slicing machine (Leica UC7). After staining with uranyl acetate and lead citrate, the samples were imaged using a transmission electron microscope (Hitachi HT7700).

### Virulence assay

*A. fumigatus* virulence assays in *Galleria mellonella* were carried out as previously described (77, 78) with minor modifications. Briefly, *G. mellonella* larvae were injected through the last prolegs with 10 µL of PBS containing 6 × 10^7^ conidia of the respective strains. The larvae only injected with 10 µL of PBS served as a control group. All larvae were incubated at 37°C in the dark, and their survival was monitored every 24 h for 7 days. Three biological repetitions were performed, and each group had 20 larvae.

### Data analysis

All statistical analyses were performed using GraphPad Prism 8 software. Multiple comparisons were analyzed by one-way analysis of variance. A *P*-value of 0.05 was considered statistically significant.

## Data Availability

The RNA-seq data have been deposited in the NCBI Sequence Read Archive under accession number PRJNA1007789. Other relevant data supporting the findings of this study are available in this article and its associated supplemental material.

## SUPPLEMENTAL MATERIAL

Supplemental table and figures msphere.00619-23-s0001.docx Table S1; Figures S1-S6.

Table S2 msphere.00619-23-s0002.xlsx RNA-seq data sets.

Table S3 msphere.00619-23-s0003.doc Strain used in this study.

Table S4 msphere.00619-23-s0004.docx Primers used in this study.

ASM does not own the copyrights to Supplemental Material that may be linked to, or accessed through, an article. The authors have granted ASM a non-exclusive, world-wide license to publish the Supplemental Material files. Please contact the corresponding author directly for reuse.
