# Supplementary material for: The basic leucine zipper transcription factor MeaB is critical for biofilm formation, cell wall integrity, and virulence in Aspergillus fumigatus
Source: mSphere. 2024 Jan 29;9(2):e00619-23. doi: 10.1128/msphere.00619-23 (PMC10900910; doi:10.1128/msphere.00619-23)
Supplement: Supplemental table and figures — Table S1; Figures S1-S6. [file msphere.00619-23-s0001.docx]

**Table S1 Transcription factors identified by SomA ChIP-seq and RNA-seq**

| **Fungi DB**  **Gene ID** | **Description** | **Fold change**  **(*somA* mutant/control)** | **TF null mutants**  **library ID** |
| --- | --- | --- | --- |
| AFUB_001820 | C2H2 finger domain protein | 1.28 | 5E6 |
| AFUB_004210 | C6 transcription factor, putative | 0.76 | 1A7 |
| AFUB_011800 | jumonji family transcription factor | 1.22 | 1C3 |
| AFUB_012530 | C2H2 transcription factor | 0.86 | 1C5 |
| AFUB_014780 | C6 transcription factor, putative | 0.61 | 1D4 |
| AFUB_016450 | HLH DNA binding domain protein | 0.29 | 6A2 |
| AFUB_019160 | C2H2 finger domain protein | 1.48 | 5E11 |
| AFUB_027530 | C2H2 transcription factor CreA | 1.20 | 1G11 |
| AFUB_030360 | CCAAT-binding transcription factor HapB | 0.91 | 1H5 |
| AFUB_031910 | C2H2 finger domain protein, putative | 0.35 | 5F4 |
| AFUB_035140 | C2H2 finger domain protein | 0.26 | 5F7 |
| AFUB_037850 | bZIP transcription factor AtfA | 1.14 | 2B7 |
| AFUB_038200 | bZIP transcription factor MeaB | 0.37 | 2B9 |
| AFUB_041060 | C6 transcription factor OTam | 0.58 | 2C6 |
| AFUB_041100 | C2H2 transcription factor Ace1 | 0.69 | 2C7 |
| AFUB_044670 | fungal specific transcription factor | 0.09 | 2D7 |
| AFUB_046410 | C6 transcription factor Ctf1B-like | 1.80 | 2E7 |
| AFUB_051340 | C6 transcription factor, putative | 0.76 | 2F11 |
| AFUB_051950 | PHD transcription factor Rum1 | 0.89 | 2G2 |
| AFUB_055550 | HLH DNA binding protein Penr2 | 1.01 | 6A3 |
| AFUB_056530 | transcription factor RfeG, putative | 0.24 | 2G12 |
| AFUB_066180 | C2H2 transcription factor Seb1 | 0.12 | 3B2 |
| AFUB_067230 | homeobox transcription factor | 1.35 | 3B5 |
| AFUB_067240 | C6 finger domain protein | 0.23 | 5C11 |
| AFUB_067320 | transcription factor RfeF, putative | 0.93 | 3B7 |
| AFUB_067440 | C6 finger domain protein | 0.33 | 5C12 |
| AFUB_069420 | bZIP transcription factor CpcA | 0.72 | 3B11 |
| AFUB_095620 | C2H2 finger domain protein | 0.72 | 5H9 |
| AFUB_099590 | HLH DNA binding domain protein | 1.24 | 6A5 |


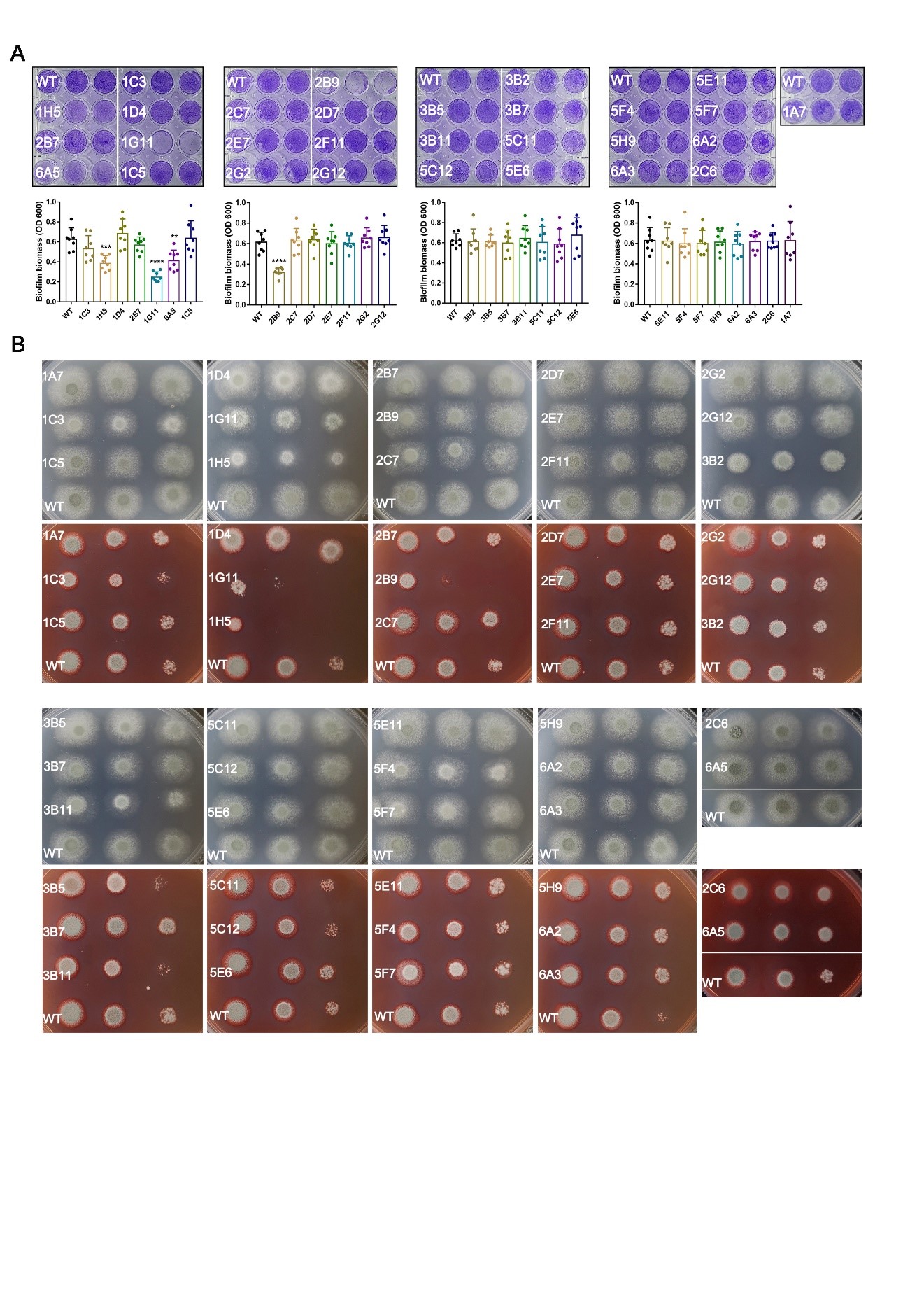


FIG S1 Identification of *A. fumigatus* transcription factors play a role in biofilm formation and cell wall integrity. (A) Formation of adherent biofilms by the WT and Δ*meaB* and *meaB^com^* strains after 24 h of growth on polystyrene plate surfaces in AMM. Biofilms were visualized by staining with crystal violet and quantified by determining the absorbance at 600 nm. (B) Phenotypes of the WT and transcription factors null mutants cultured on AMM supplemented with CR at 37°C. Colony morphology was imaged after 48 h. The indicated gene IDs can be found in the Table S1.


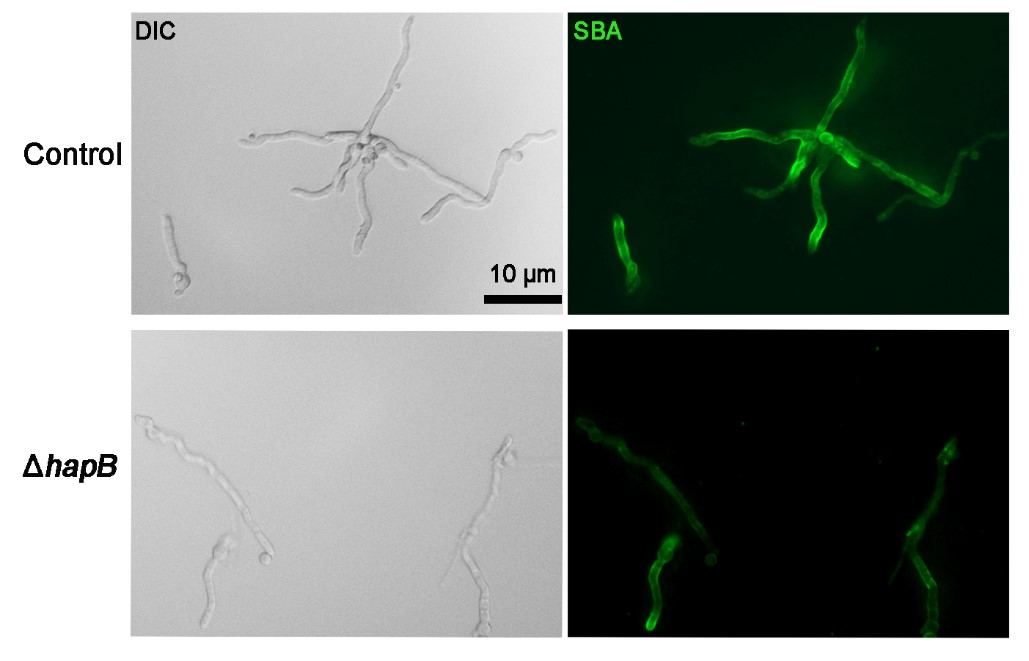


FIG S2 Representative images of hyphae of the WT and Δ*hapB* strains stained with SBA-FITC after growth in RPMI 1640. Scale bar, 10 μm. DIC, differential interference contrast.


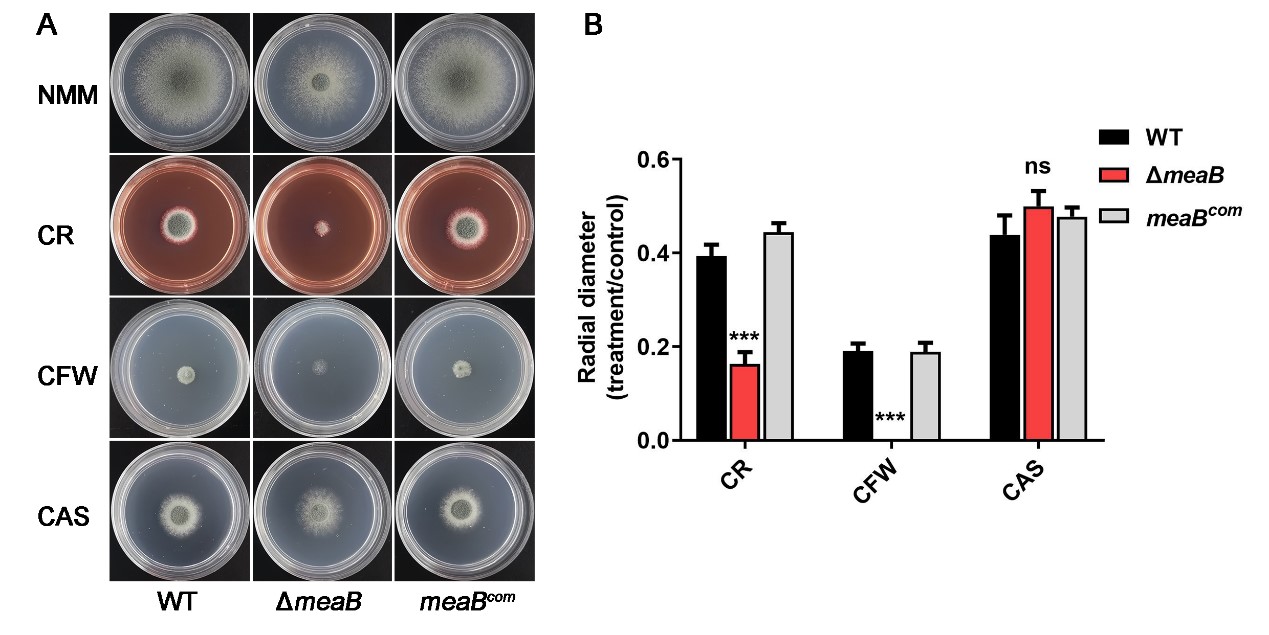


FIG S3 (A) Phenotypes of the WT and Δ*meaB* and *meaB^com^* strains cultured on NMM supplemented with CR, CFW, and CAS at 37°C. Colony morphology was imaged after 48 h. (B) Quantitative analysis of relative colony diameter of the WT and Δ*meaB* and *meaB^com^* strains. The data are presented as the ratio of treatment to control groups of the indicated strain, and the results are the means of three repetitions ± SD. (***, *P* < 0.001; ns, not significant).


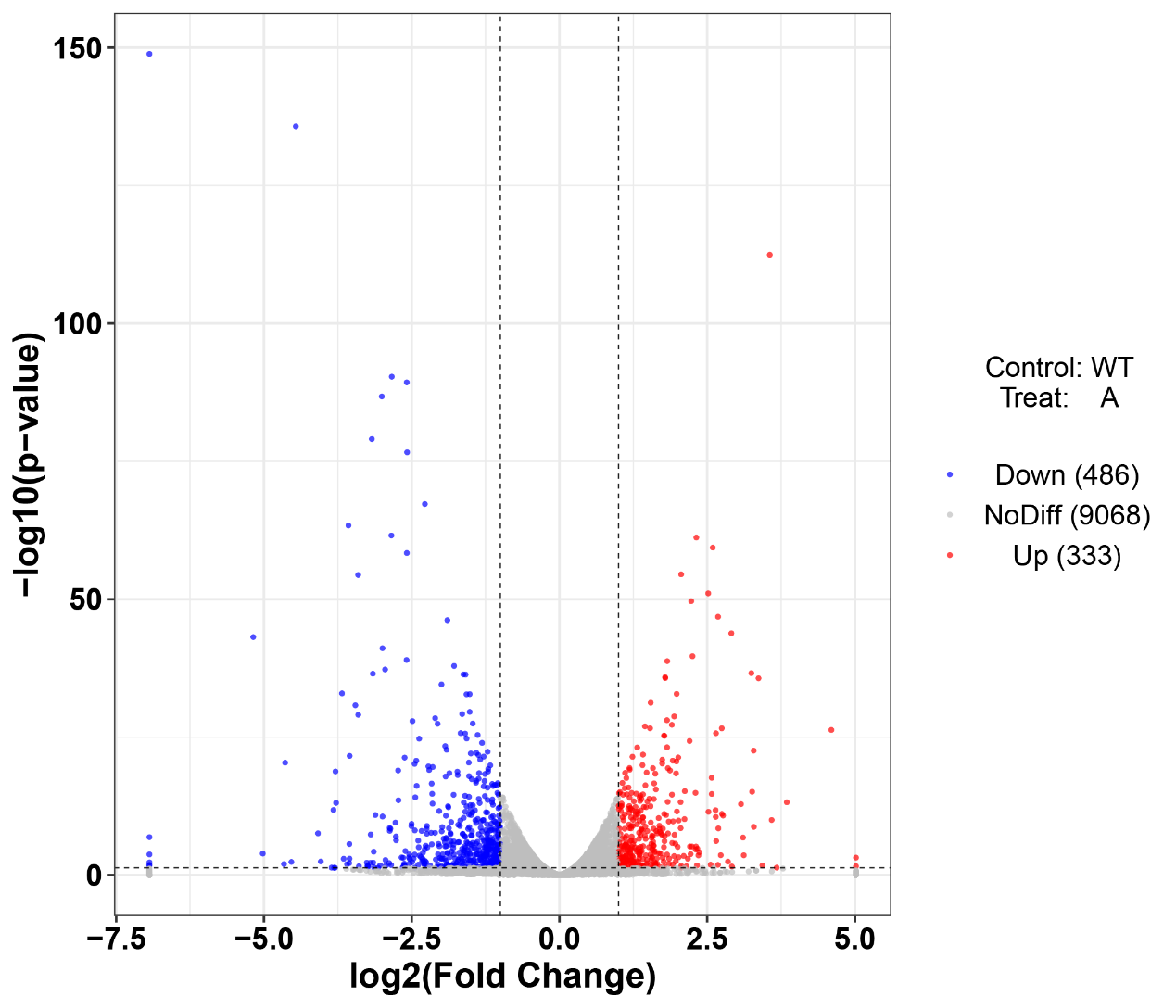


FIG S4 Volcano map shows the differentially expressed genes in the Δ*meaB* mutant strain.


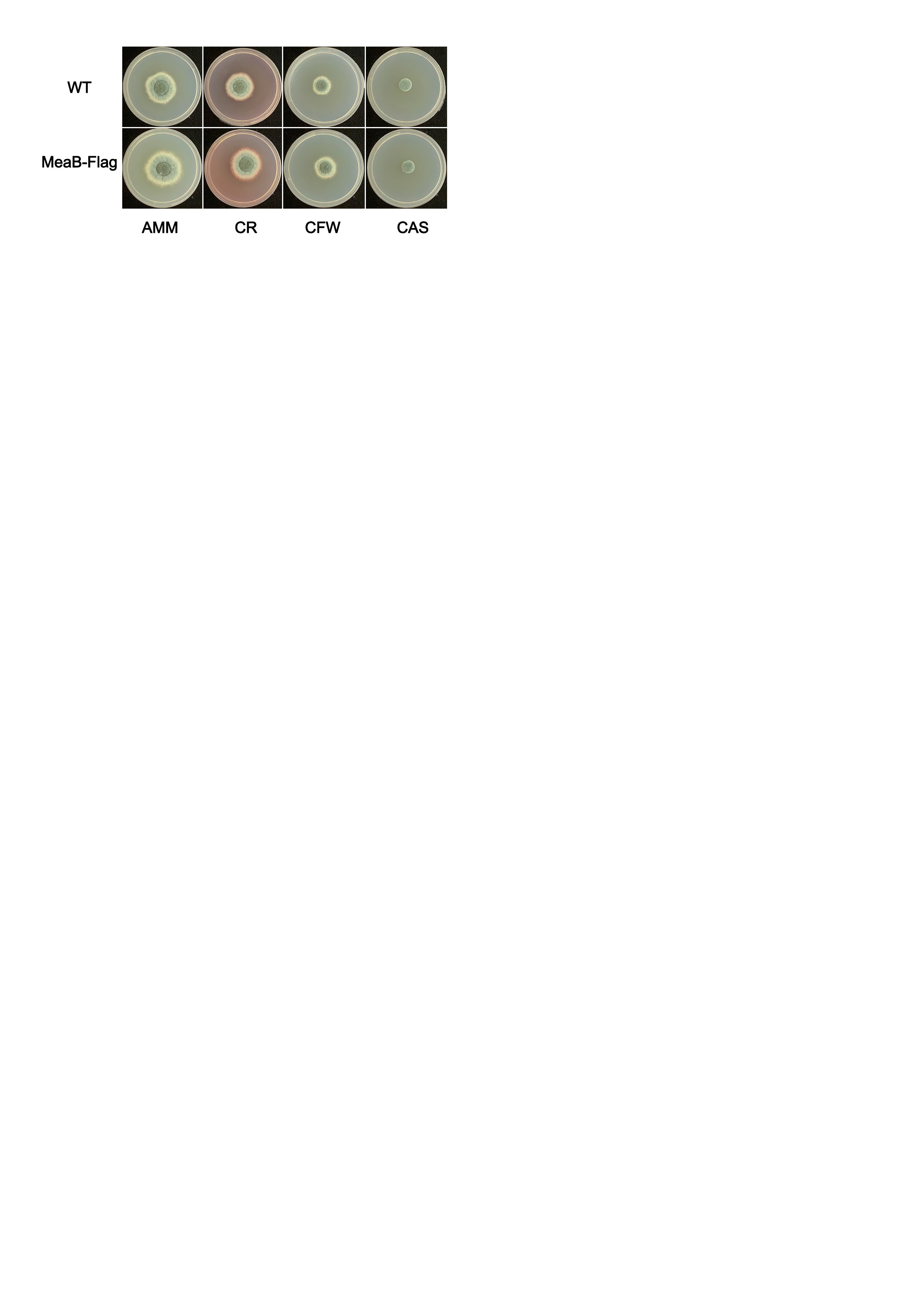


FIG S5 Phenotypes of the WT and MeaB-Flag strains cultured on AMM supplemented with CR, CFW, and CAS at 37°C. Colony morphology was imaged after 48 h.


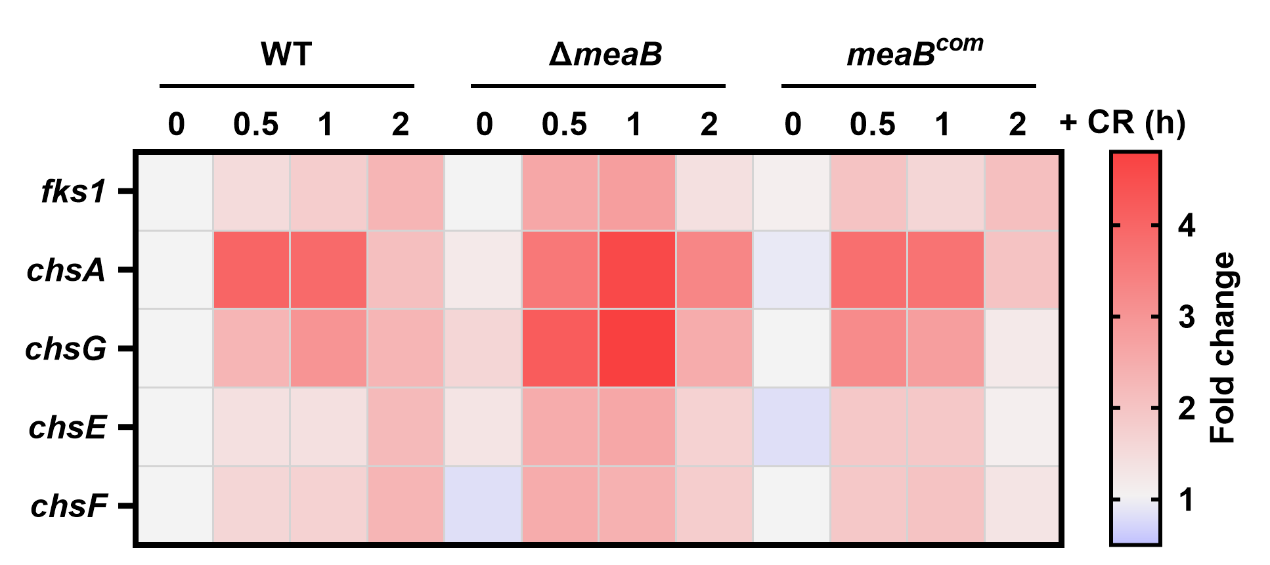


FIG S6 Heat map shows the relative expression levels of cell wall biosynthesis related-genes in the WT and Δ*meaB* and *meaB^com^* strains in the presence of 300 μg/ml CR for 0.5, 1, or 2 h. Gene expression was normalized to the endogenous reference gene *tubA*. Results represent data from three independent biological experiments.
