# Supplementary material for: The basic leucine zipper transcription factor MeaB is critical for biofilm formation, cell wall integrity, and virulence in Aspergillus fumigatus
Source: mSphere. 2024 Jan 29;9(2):e00619-23. doi: 10.1128/msphere.00619-23 (PMC10900910; doi:10.1128/msphere.00619-23)
Supplement: Table S3 — Strain used in this study. [file msphere.00619-23-s0003.doc]

**Table S3. S**trains used in this study

| **Strain** | **Genotype** | **Reference** |
| --- | --- | --- |
| A1160 | Δ*ku80, pyrG* | FGSC |
| 1160^C^ (WT) | *A1160, pyr4* | [1] |
| ∆*meaB* | ∆*ku80, pyrG,* ∆*meaB::pyr4* | This study |
| *meaB^Com^* | ∆*ku80, pyrG,* ∆*meaB::pyr4, meaB (P):: meaB::hph* | This study |
| MeaB-FLAG | ∆*ku80, pyrG, meaB::5×FLAG:: pyr4* | This study |
| ∆*HapB* | Δ*ku80*; *pyrG1*; *AMA1::P_gpdA_::Cas9::pyr4*; Δ*hapB::hph* | [2] |

**References**

1. Bertuzzi M, van Rhijn N, Krappmann S, Bowyer P, Bromley MJ, Bignell EM. 2021. On the lineage of Aspergillus fumigatus isolates in common laboratory use. Medical Mycology 59:7-13.

2. Ren, Y.; Zhang, C.; Chen, Z.; Lu, L.; Fischer, R. The Heterotrimeric Transcription Factor CCAAT-Binding Complex and Ca2+-CrzA Signaling Reversely Regulate the Transition between Fungal Hyphal Growth and Asexual Reproduction. *mBio* **2021**, *12*, doi:10.1128/mBio.03007-21.
