# Supplementary material for: The basic leucine zipper transcription factor MeaB is critical for biofilm formation, cell wall integrity, and virulence in Aspergillus fumigatus
Source: mSphere. 2024 Jan 29;9(2):e00619-23. doi: 10.1128/msphere.00619-23 (PMC10900910; doi:10.1128/msphere.00619-23)
Supplement: Table S4 — Primers used in this study. [file msphere.00619-23-s0004.docx]

**Table S4.** **Primers used in this study**

| **Name/Purpose** | **Sequence (5’ to 3’)** |
| --- | --- |
| **Genes deletion and complementation** | |
| MeaB-P1 | ATCTCAGAACCTGAGCAG |
| MeaB-P2 | CTAAGTCCACCTGTTGCCT |
| MeaB-P3 | CGATTAAGTTGGGTAACGCCAGTCTCGACCATAGAGACCAT |
| MeaB-P4 | ATAAGTAGCCAGTTCCCCATTCTCGTCATTAAGCGTC |
| MeaB-P5 | CACTCGTTTCGCACACATAC |
| MeaB-P6 | GTCGATCGATAGCTCGAAC |
| MeaB-SF | TCGACCAGTTCATCAGTGTC |
| MeaB-SR | AATGCCGTATGTCCCATCAC |
| meaB^com^ -F | GAGGTAATCCTTCTTTCTAGAGACGTTCCAGAACTGCACATC |
| meaB^com^ -R | ACGACGGCCAGTGCCAAGCTTTAGAGCTGTCATGTGGCCAGAG |
| Pyr4F | TGGCGTTACCCAACTTAATCG |
| Pyr4R | GCTTTCGGGAACTGGCTACTTAT |
| cPyr4R | GGTCCAAGTGGAAGTAGGTAGTGAC |
| Cpyr4F | GGAATAAGAAATGGCGGAGGAT |
| **MeaB-FLAG construction and confirmation** | |
| MeaBflag-P1 | TCGACCAGTTCATCAGTGTC |
| MeaBflag-P2 | GCAACGTAACAGACAAGCTCAG |
| MeaBflag-P3 | CATTCCCGGGGATCCCTCGAGATGACGAGAATGGTTTTGCTC |
| MeaBflag-P4 | ATAAGTAGCCAGTTCCCGAAAGCTAAGCGTCGCTGTCCACTTCA |
| MeaBflag-P5 | CACTCGTTTCGCACACATAC |
| MeaBflag- P6 | GTCGATCGATAGCTCGAAC |
| Flag SF | CTCGAGGGATCCCCGGGAATG |
| Flag-pyr4 SR | GCTTTCGGGAACTGGCTACTTAT |
| **RT-qPCR** | |
| RTtub F | TTCCGTCCCGACAACTTCGT |
| RTtub R | TCACAGCCTTCAGCCTCACG |
| RTmeaB F | CAGAGACTTCCAGCCGGGATTG |
| RTmeaB R | TGGGCTTGTTGCGCGTCCAT |
| RThapB F | CAAATCCACAGGGACACATG |
| RThapB R | AGATACCGTATGGCTGAGGC |
| RTcreA F | CTCCCTCGACCCCATCAACT |
| RTcreA R | GTGTCTGGTCTGATGCTCCAG |
| RTgtb3 F | CACTCCTCCGTGGACTGCTT |
| RTgtb3 R | ACGGATGAAACCGCCTTGGA |
| RTega3 F | ACGACAAGTCCACCATCGCA |
| RTega3 R | GCCCAGATCCGAGTCCTTGA |
| RTsph3 F | AGAATGTGCGGCTGCTAGGC |
| RTsph3 R | CAGCTAGAGCCGGGTTGGAA |
| RTuge3 F | GCTGTTAGCCTCCCAGTACC |
| RTuge3 R | GGACTTGGTCGTACCCCAT |
| RTagd3 F | ACGCGGACGTCTTCAAGGAG |
| RTagd3 R | GTTGTGCAGACCGGTGATGG |
| RTchsA F | GGCGGGAAGGTTGACGTTGA |
| RTchsA R | AAACACCCAAGCGAGCACGA |
| RTchsE F | CCAACGGCCCGACTCTCATC |
| RTchsE R | CGCCGAGACGTGTAAAGGCA |
| RTchsF F | ACCAGGTGGGCACAGGAAGA |
| RTchsF R | TCCCTGCCTCTGTGCTCTGT |
| RTchsG F | GGGCCGTTTGATGACCCTCAG |
| RTchsG R | CGACACGTCCAGGGACACCAA |
| RTfks1 F | CCGCACGACGACTACTACGG |
| RTfks1 R | AACGTTCATCACCGCGACCA |
| RTgel1 F | TGCTGTTACTGCCGCTCTCG |
| RTgel1 R | TCAGCGATGGGATCAGCCAG |
| RTge5 F | GCAATGGTACCACTGATGGC |
| RTgel5 R | GGCCAGCATCTGCATACACTC |
| RTgel7 F | AGCAGGACTATTCCGGCAGCCA |
| RTgel7 R | ATACCGGCCTGGCTGAGAAGGT |
| RTmpkA F | GCTATCATCCGCTCGGGACA |
| RTmpkA R | TTCTCCTCGGGGTCGATCGA |
| RTmpkC F | TCCGTGCGGAGGTCTTGGGTA |
| RTmpkC R | ACGCTTCGCCAGAGACGAACTG |
| **EMSA Probe** | |
| Cy5-label | AGCACGTGGTCGAAAG |
| EMSA meaB F | AGCACGTGGTCGAAAGGTCCTGATGGGTCGTTATGTC |
| EMSA meaB R | AGCACGTGGTCGAAAGGATTGTAGCCTAGCAGGCAC |
